# Proline Accumulation in Barley Under Salinity Is ABA-Independent, but Relies on the Level of Oxidative Stress When Modulated by Mo and W Ions

**DOI:** 10.3390/ijms27021104

**Published:** 2026-01-22

**Authors:** Moldir Beisekova, Beata Michniewska, Weronika Kusek, Alua Zh. Akbassova, Rustem Omarov, Sławomir Orzechowski, Edyta Zdunek-Zastocka

**Affiliations:** 1 Department of Biotechnology and Microbiology, L.N. Gumilyov Eurasian National University, Astana 010000, Kazakhstan; mk.beisekova@gmail.com (M.B.); a.j.alua@gmail.com (A.Z.A.);; 2 Department of Biochemistry and Microbiology, Warsaw University of Life Sciences-SGGW, Nowoursynowska 159, 02-776 Warsaw, Poland; beata_michniewska@sggw.edu.pl (B.M.); weronika_kusek@sggw.edu.pl (W.K.); slawomir_orzechowski@sggw.edu.pl (S.O.)

**Keywords:** abscisic acid, aldehyde oxidase, molybdenum, oxidative stress, proline, reactive oxygen species, salinity, tungsten

## Abstract

The accumulation of proline, an important osmoprotective and antioxidant compound, is a key defense mechanism induced in plants in response to stress factors, including salinity, and is likely dependent on abscisic acid (ABA). However, in barley grown for 8 days under salinity conditions (125 mM NaCl), proline accumulation was not accompanied by changes in ABA content. Co-application of 0.5 mM molybdenum (Mo) significantly reduced NaCl-induced oxidative stress, as measured by H_2_O_2_, O_2_^−^, MDA, and chlorophyll content, and increased the activity of Mo-containing aldehyde oxidase (AO), an enzyme involved in de novo ABA synthesis. As a result, elevated ABA levels were observed, but proline content under salinity conditions was similar in Mo-treated and non-Mo-treated plants. In contrast, exposing plants to 0.5 mM tungsten (W), an antagonist of Mo, inhibited AO activity without significantly altering ABA content, while proline and oxidative stress marker levels increased dramatically under both non-saline and saline conditions. The observed changes in proline content are mainly due to modulation of the rate of synthesis and, to a lesser extent, the rate of degradation, as revealed by transcript abundance of *P5CS1* and *PDH*, which encode D1-pyrroline-5-carboxylate synthetase and proline dehydrogenase, respectively. The results indicate that in barley grown under salinity conditions, proline accumulation is ABA-independent but depends on the level of oxidative stress modulated by Mo and W ions.

## 1. Introduction

Plants constantly face harsh environmental conditions such as salinity, drought, severe temperatures, UV radiation, or heavy metal contamination, which negatively affect plant growth and development, thereby reducing crop yields [1]. Among abiotic stress factors, high salt concentrations, mainly sodium and chloride ions, are one of the most serious environmental problems worldwide. Soil salinization limits water uptake by plants and causes Na^+^ and Cl^−^ ions to accumulate to toxic levels, ultimately leading to osmotic and ionic stress [2,3]. Excessive uptake of toxic ions disrupts various metabolic processes, including photosynthesis and nitrogen assimilation, and causes excessive production of reactive oxygen species (ROS), leading to oxidative damage of functional proteins, nucleic acids, and membrane lipids [4].

To overcome the negative effects of stress factors, plants have developed various defense strategies, among which the accumulation of organic osmolytes such as proline, glycine betaine, or trehalose, is one of the most common and widespread [5,6]. Proline is a multifunctional amino acid that plays a key role in osmotic adjustment, maintaining cell turgor pressure, and stabilizing cellular structures such as proteins and cell membranes [7]. In addition, proline acts as a signaling molecule, participates in the antioxidant response, and serves as a nitrogen and carbon source for rapid recovery from stress [8,9]. Accordingly, exogenous proline administration improves osmoregulation, reduces oxidative damage, and consequently increases biomass production and crop yields under various stress conditions [10,11,12,13].

In plants, proline synthesis occurs in the cytoplasm and chloroplast via two pathways: the glutamate pathway and the ornithine pathway, with the former typically dominating in stress-induced proline synthesis [7,14,15,16]. In this pathway, glutamate is reduced by the rate-limiting D1-pyrroline-5-carboxylate synthase (P5CS) to glutamate semialdehyde, which is then spontaneously converted to pyrroline-5-carboxylate (P5C). Finally, P5C reductase (P5CR) reduces P5C to proline. In turn, proline catabolism occurs in mitochondria through the oxidation of proline to P5C catalyzed by proline dehydrogenase (PDH), followed by the conversion of P5C to glutamate by P5C dehydrogenase (P5CDH) [16]. It is commonly accepted that under adverse conditions, the rate-limiting enzymes in proline biosynthesis and degradation are P5CS and PDH, respectively, and their activity is regulated at the transcriptional level [15,16,17]. In *Arabidopsis thaliana* and *Pisum sativum*, P5CS is encoded by two genes, with transcription of *AtP5CS1* and *PsP5CS2* being strongly induced under drought, salinity, and Cd toxicity, respectively [18,19]. In wheat and barley, only one *P5CS* gene has been characterized so far, whose transcriptional up-regulation precedes proline accumulation under drought and salinity [20,21,22]. In turn, the expression of *PDH1* and *PDH2* in *Medicago sativa* and *PDH1* in Arabidopsis decreased significantly under salinity conditions [23,24], while a mutation in the single-copy *OsPDH* gene in rice caused an increase in proline content and increased heat stress tolerance [25].

Although the accumulation of endogenous proline in response to osmotic stress is well documented, the signaling mechanisms by which environmental stresses regulate proline metabolism remain poorly understood, especially in agricultural crops. In Arabidopsis, increased expression of *P5CS1* under salinity may be induced by phospholipase C and calcium signaling [26] or may involve phytohormones such as abscisic acid (ABA) [27,28]. In barley, mutation in the ABA-responsive element (ABRE) of the *P5CS1* promoter significantly reduces *P5CS1* expression and proline accumulation in response to drought [29], highlighting the ABA-dependent nature of proline accumulation. However, studies using Arabidopsis ABA-deficient mutants (*aba1-1* and *aba2-1*) have shown that the regulation of proline biosynthesis upon cold and osmotic stress occurs via an ABA-independent pathway [30,31]. Additionally, studies using fluridone, an inhibitor of ABA synthesis, showed that in wilted barley leaves ABA accumulation is not required for proline accumulation [32], indicating that the signaling mechanisms regulating proline metabolism under stress largely remain to be elucidated.

In plants, de novo ABA synthesis begins in chloroplasts with the cleavage of a C_40_ 9-*cis*-epoxycarotenoid precursor [33]. The final step of this pathway is the oxidation of abscisic aldehyde to ABA, which occurs in the cytosol and is catalyzed by the aldehyde oxidase (AO) [34]. An increase in AO activity was observed in response to ammonium treatment or tombusvirus infection in barley [35,36], as well as in response to drought, salinity, nitrogen deficiency, and Cd toxicity in pea plants, and was accompanied by an increase in ABA concentration [19,37,38]. AO belongs to a family of molybdoenzymes that require a specific form of organic molybdenum, known as the molybdenum cofactor (Moco), for their catalytic activity [39,40].

Molybdenum (Mo) is one of the trace elements essential for plant growth and development [41]. In plants, as a component of Moco of AO, nitrate reductase (NR), xanthine dehydrogenase (XDH), sulfite oxidase (SO), and amidoxime reducing component (ARC), it is crucial not only for plant hormone synthesis, but also for nitrogen assimilation, purine catabolism, and sulfur metabolism [42,43,44]. Recently, it has been shown that adequate Mo application increases crop yields and improves plant tolerance to stress caused by drought, salinity, cold, or Cd [45,46,47,48], although the mechanisms underlying Mo-derived stress tolerance are not fully understood. Under Cd toxicity, Mo alleviates oxidative stress in rice by restricting Cd uptake and strengthening the antioxidant defense system, mainly through the activation of ROS-detoxifying enzymes [49]. Under drought conditions in wheat, Mo increases relative water content, chlorophyll content, photosynthesis rate, and accumulation of K^+^ ions, activates antioxidant defense, and improves osmotic adaptation capacity by increasing the concentration of soluble sugars and proteins [46].

To clarify the mechanism by which Mo activates defense responses to salinity, we hypothesized that Mo may enhance stress tolerance by inducing AO-dependent ABA biosynthesis, which subsequently leads to ABA-dependent proline accumulation. Therefore, we measured the effects of Mo and tungsten (W) application on oxidative stress markers, AO activity, ABA content, proline content, and the expression of its major metabolic genes in barley plants grown under non-saline and saline conditions. Tungsten, considered an antagonist of Mo and capable of replacing it in the center of Moco [50,51], may inhibit AO activity and stress-induced ABA synthesis, allowing us to verify whether proline accumulation can occur independently of ABA accumulation. Here, we report that W supplementation induces oxidative stress and proline synthesis and accumulation, which occur without ABA accumulation, both under control and salt stress conditions. In contrast, Mo application increased the synthesis and content of both ABA and proline under control conditions and maintained these elevated levels under salinity, which may contribute to Mo-induced alleviation of NaCl-induced oxidative stress.

## 2. Results

### 2.1. Biomass Production as Affected by Salinity and Mo or W Treatment

**Figure 1:**
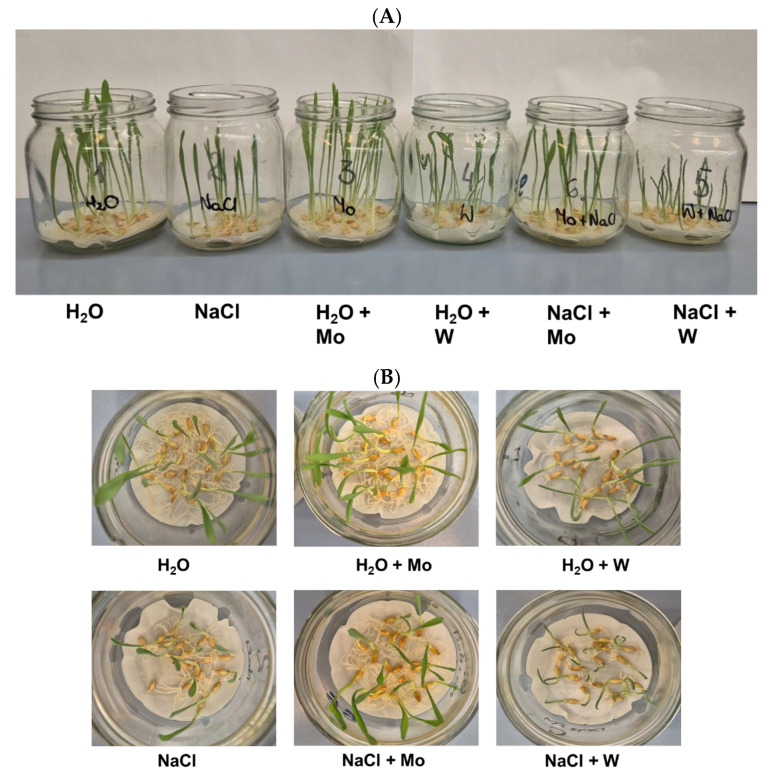
The effect of molybdenum (Mo) and tungsten (W) on the appearance of shoots ((**A**), side view) and roots ((**B**), top view) of 8-day-old barley grown under salt-free and salt stress conditions. Mo was applied as 0.5 mM Na_2_MoO_4_, W was applied as 0.5 mM Na_2_WO_4_, and salinity stress was induced with 125 mM NaCl. The images represent plants from one of three independent biological replicates, yielding similar results.

**Figure 2:**
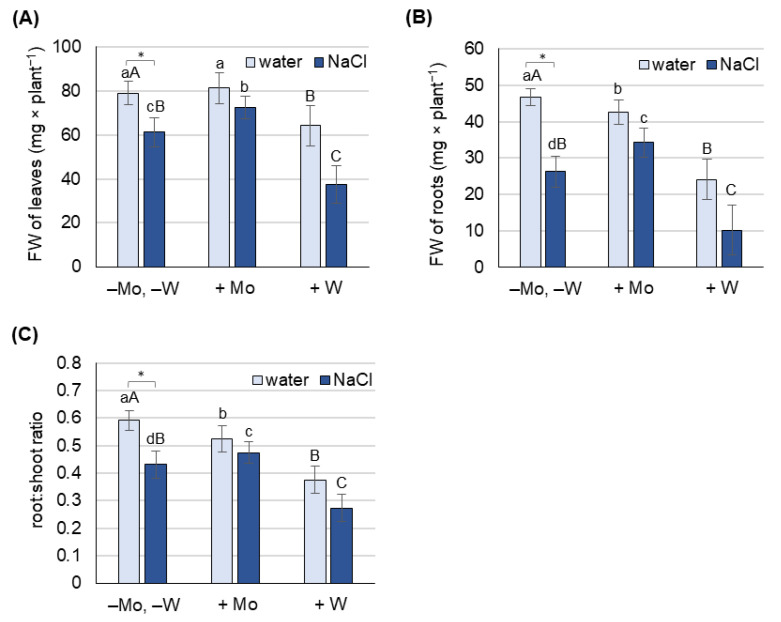
The effect of molybdenum (Mo) and tungsten (W) on the fresh weight (FW) in leaves (**A**) and roots (**B**), as well as on the root-to-shoot ratio (**C**), of 8-day-old barley grown under non-saline and salinity stress conditions. Mo was applied as 0.5 mM Na_2_MoO_4_, W as 0.5 mM Na_2_WO_4_, and salinity stress was induced with 125 mM NaCl. Results are means (±SD) of three biological replicates. Significant differences (at least *p* < 0.05) between means for plants grown only on water (−Mo, −W) and those grown on NaCl solution (−Mo, −W, +NaCl) are marked with asterisks. Significant differences (at least *p* < 0.05) between means for plants not treated with Mo or W (−Mo, −W) and those treated with Mo (+Mo) are indicated above the columns with different lowercase letters, while significant differences compared to plants treated with W (+W) are indicated with different uppercase letters.

The effect of Mo and W treatment on appearance and biomass production was studied in 8-day-old barley grown under non-saline and salt stress conditions, separately for leaves (Figure 1A and Figure 2A) and roots (Figure 1B and Figure 2B).

Under non-saline conditions, Mo treatment did not significantly change the fresh weight of barley leaves but reduced the fresh weight of roots by about 10%. In contrast, W treatment reduced the fresh weight of both leaves and roots by 25% and 50%, respectively.

Salt stress decreased the fresh weight of barley grown on water (−Mo, −W) by 25% for leaves and 45% for roots. Under salinity, Mo treatment increased the fresh weight of both leaves and roots by 12% and 30%, respectively. In contrast, W treatment further reduced the fresh weight of both leaves and roots by 35% and 55%, respectively.

### 2.2. Concentration of Oxidative Stress Markers and Chlorophyll as Affected by Salinity and Mo or W Treatment

**Figure 3:**
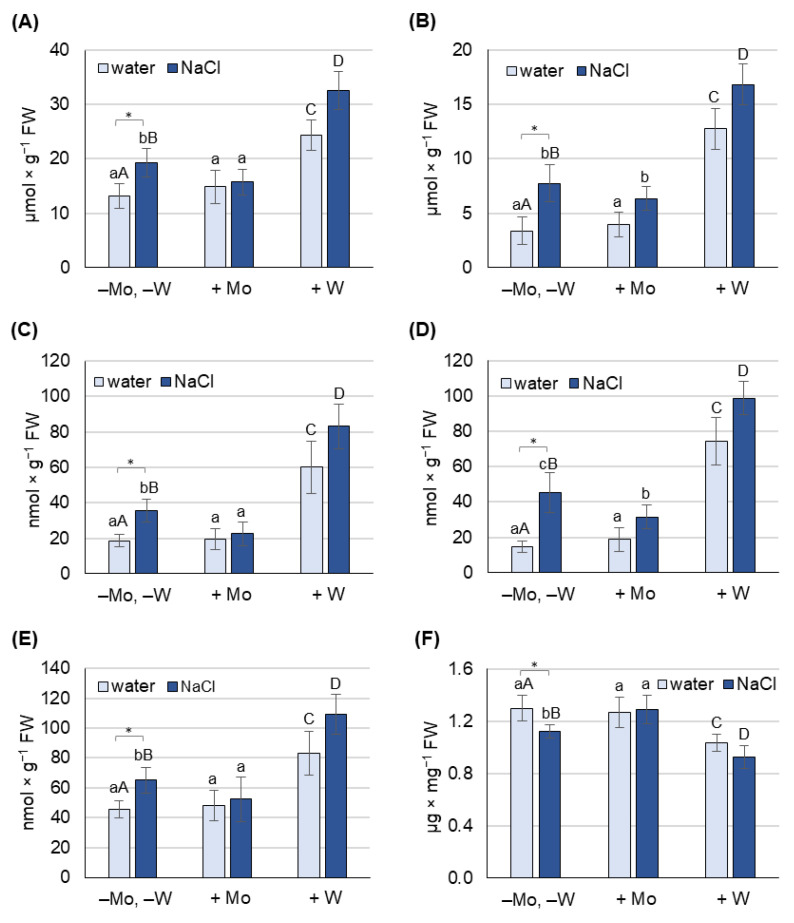
The effect of molybdenum (Mo) and tungsten (W) on H_2_O_2_ (**A**,**B**), O_2_^−^ (**C**,**D**), MDA (**E**), and chlorophyll (**F**) concentrations in leaves (**A**,**C**–**F**) and roots (**B**,**D**) of 8-day-old barley grown under non-saline and salinity stress conditions. Mo was applied as 0.5 mM Na_2_MoO_4_, W as 0.5 mM Na_2_WO_4_, and salinity stress was induced with 125 mM NaCl. Results are means (±SD) of three biological replicates. Significant differences (at least *p* < 0.05) between the means for plants grown on water only (−Mo, −W) and those grown on water and NaCl (−Mo, −W, +NaCl) are marked with asterisks. Significant differences (at least *p* < 0.05) between the means for plants not treated with Mo or W (−Mo, −W) and those treated with Mo (+Mo) are indicated above the columns with different lowercase letters, while significant differences compared to plants treated with W (+W) are marked with different uppercase letters.

The concentrations of ROS (H_2_O_2_ and O_2_^−^) and indicators of cell membrane damage (MDA, chlorophyll) caused by Mo and W were determined in 8-day-old barley grown under non-saline and salt stress conditions, separately for leaves (Figure 3A,C,E,F) and roots (Figure 3B,D).

Under non-saline conditions, Mo application did not cause significant changes in any of the oxidative stress indicators examined in either leaves or roots. Molybdenum also had no effect on chlorophyll concentration. In turn, treatment with W increased all measured oxidative stress indicators in both leaves and roots and reduced chlorophyll content. Tungsten increased the concentration of H_2_O_2_ in leaves and roots by 85% and 300%, respectively, and the concentration of O_2_^−^ by 220% and 400%, respectively. In leaves, W increased the concentration of MDA by 80% and decreased chlorophyll content by 20%.

Salt stress in barley grown on water (−Mo, −W) increased all measured oxidative stress indicators in both leaves and roots and resulted in a 15% decrease in chlorophyll content in leaves. In these plants, salinity increased H_2_O_2_ concentration by 50% in leaves and 130% in roots, O_2_^−^ concentration by 90% in leaves and 200% in roots, and MDA concentration by 40%.

Under saline conditions, the application of Mo reduced the concentration of all examined oxidative stress indicators in both leaves and roots. Mo reduced H_2_O_2_ concentration by 20%, O_2_^−^ concentration by 35%, and MDA concentration by 20%. Mo also increased chlorophyll concentration by 15%, restoring it to the level of plants grown solely on water (control group).

Under salinity conditions, treatment with W caused a further increase in the concentration of all oxidative stress indicators tested in both leaves and roots. Tungsten increased H_2_O_2_ concentration by 70% in leaves and 120% in roots, O_2_^−^ concentration by 120% in both leaves and roots, and MDA concentration by 70%. Treatment with W also caused a further 20% decrease in chlorophyll content.

Based on the results obtained, it can be concluded that treating the plants with Mo alleviated the oxidative stress caused by salinity, while treating the plants with W further exacerbated oxidative damage.

### 2.3. Proline Concentration and Its Metabolism as Affected by Salinity and Mo or W Treatment

**Figure 4:**
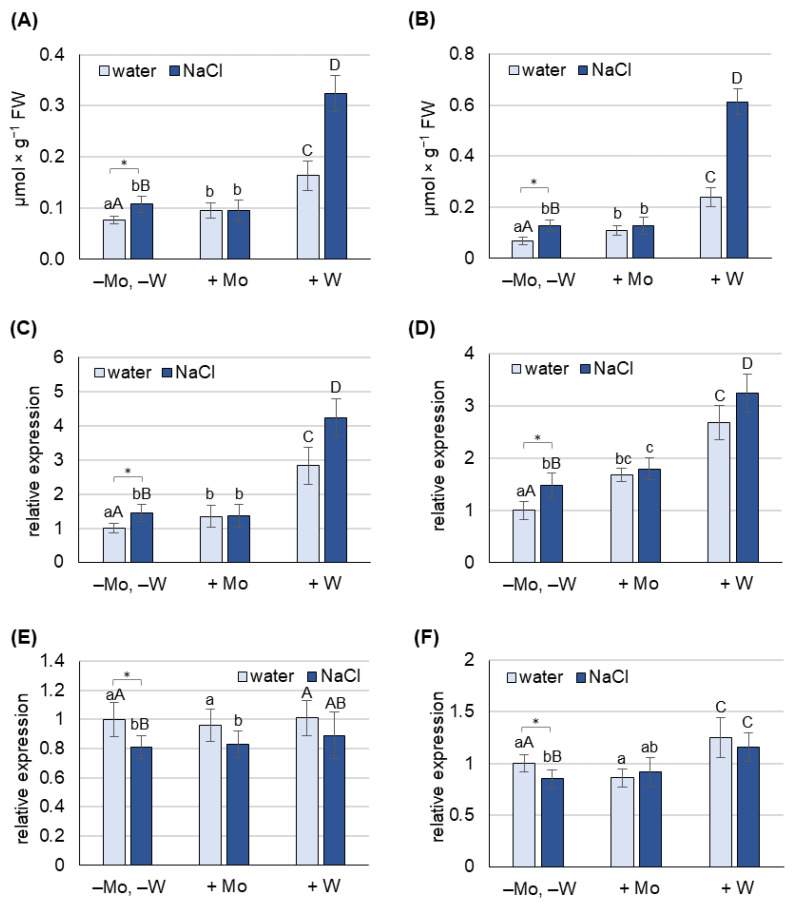
The effect of molybdenum (Mo) and tungsten (W) on proline content (**A**,**B**) and transcript levels of *P5CS1* (**C**,**D**) and *PDH* (**E**,**F**) in leaves (**A**,**C**,**E**) and roots (**B**,**D**,**F**) of 8-day-old barley grown under non-saline and salinity stress conditions. Mo was applied as 0.5 mM Na_2_MoO_4_, W as 0.5 mM Na_2_WO_4_, and salinity stress was induced with 125 mM NaCl. The relative mRNA level was expressed relative to that of plants grown only on water, which was set to 1 after normalization to the reference genes. Results are means (±SD) of three biological replicates. Significant differences (at least *p* < 0.05) between means for plants grown only on water (−Mo, −W) and those grown on water and NaCl (−Mo, −W, +NaCl) are marked with asterisks. Significant differences (at least *p* < 0.05) between the means for plants not treated with Mo or W (−Mo, −W) and those treated with Mo (+Mo) are marked above the columns with different lowercase letters, while significant differences compared to plants treated with W (+W) are marked with different uppercase letters.

The effect of Mo and W treatments on proline concentration and the expression of genes involved in its metabolism (*P5CS1* and *PDH*) was studied in 8-day-old barley grown under non-saline and salt stress conditions, separately for leaves (Figure 4A,C,E) and roots (Figure 4B,D,F).

Under non-saline conditions, both Mo and W treatments increased proline concentration in leaves and roots. Mo application increased proline content by 40% in leaves and 60% in roots, while W increased it by 100% in leaves and 250% in roots. This increase was accompanied by higher *P5CS1* expression: 35% in leaves and 70% in roots with Mo treatment, and 180% in both leaves and roots with W treatment.

Salt stress in barley grown on water (−Mo, −W) caused a 30% increase in proline concentration in leaves and a 90% increase in roots. This increase in proline content was accompanied by a 45% increase in *P5CS1* expression and a 15% decrease in *PDH* expression both in leaves and roots.

Under saline conditions, the application of Mo did not significantly change the concentration of proline in either leaves or roots compared to non-Mo-treated plants, while W treatment increased it by further 220% in leaves and 250% in roots. The observed increase of proline content in W-treated plants was accompanied by an increase in the expression of *P5CS1* by 190% in leaves and 120% in roots.

Based on the results obtained, it can be concluded that under non-saline conditions, treatment of plants with Mo or W increased the concentration of proline in both leaves and roots; however, the increase observed in W-treated plants was more pronounced. In turn, simultaneous treatment with NaCl and W or Mo caused a further increase in proline content only in plants treated with W, and the observed changes in proline concentration were mainly due to an increased biosynthesis rate. Therefore, mechanisms other than an increase in proline concentration are likely responsible for the alleviation of salt-induced oxidative stress by molybdenum.

### 2.4. ABA Concentration as Affected by Salinity and Mo or W Treatment

In the next stage, we examined the concentration of ABA to answer two questions: Does Mo alleviate salt-induced oxidative stress by increasing ABA concentration, and are the changes in proline concentration observed above (Figure 4A,B) related to changes in ABA concentration?

**Figure 5:**
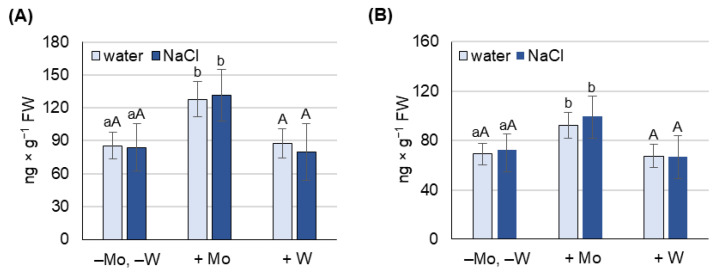
The effect of molybdenum (Mo) and tungsten (W) on ABA concentration in leaves (**A**) and roots (**B**) of 8-day-old barley grown under non-saline and salinity stress conditions. Mo was applied as 0.5 mM Na_2_MoO_4_, W as 0.5 mM Na_2_WO_4_, and salinity stress was induced with 125 mM NaCl. Results are the means (±SD) of three biological replicates. Significant differences (at least *p* < 0.05) between the means for plants not treated with Mo or W (−Mo, −W) and those treated with Mo (+Mo) are marked above the columns with different lowercase letters, while significant differences compared to plants treated with W (+W) are marked with different uppercase letters.

The effect of Mo and W treatment on ABA concentration was studied in 8-day-old barley grown under non-saline and under salt stress conditions, separately for leaves (Figure 5A) and roots (Figure 5B).

Under both non-saline and saline conditions, only the application of Mo significantly changed the concentration of ABA. Molybdenum increased ABA concentration by 50% in leaves and by 40% in roots under both growth conditions.

**Figure 6:**
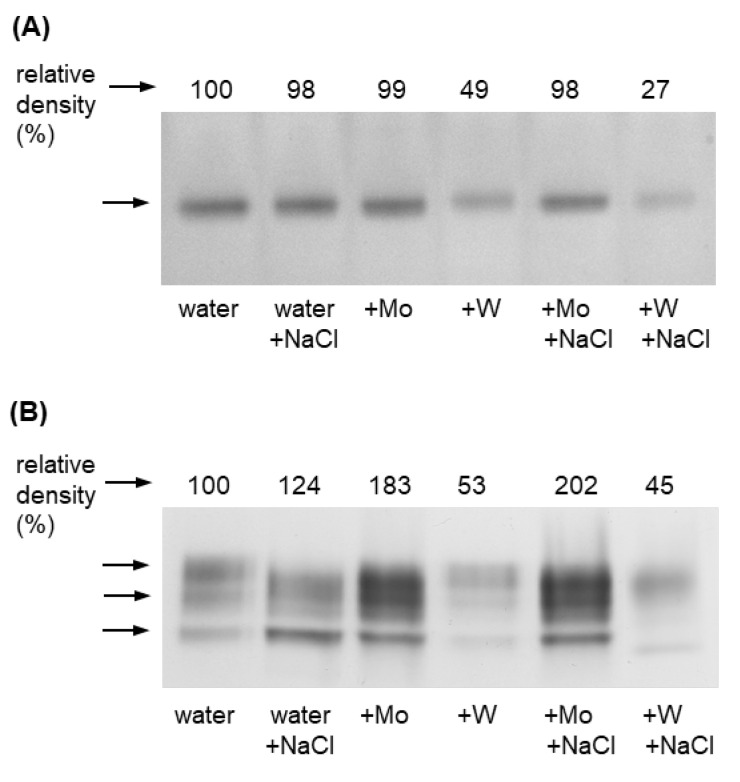
Zymograms of aldehyde oxidase from leaves (**A**) and roots (**B**) of 8-day-old barley grown under salinity stress and treated with Mo or W. Salinity stress was induced with 125 mM NaCl, Mo was applied as 0.5 mM Na_2_MoO_4_, and W as 0.5 mM Na_2_WO_4_. AO activity was assessed after native PAGE using indole-3-aldehyde as the substrate. Each lane of the gel was loaded with 100 μg of leaf proteins or 50 μg of root proteins. The numbers above the zymograms indicate relative values obtained by densitometric scanning and analysis with ImageJ software version 1.53. The zymograms shown are representative of similar results from three independent biological experiments.

Under salinity stress, an increase in ABA concentration was not observed in plants grown on water (−Mo, −W), although it cannot be ruled out that such an increase might occur in a longer-term experiment, as indicated by the increase in AO activity in roots observed at that time (Figure 6B).

The results obtained allow us to conclude, first, that Mo can alleviate salt-induced oxidative stress by increasing ABA concentration and, second, that the observed increase in proline concentration under saline conditions and after treatment with W may occur in the absence of ABA accumulation.

### 2.5. Aldehyde Oxidase Activity as Affected by Salinity and Mo or W Treatment

To confirm that the observed changes in ABA concentration are associated with changes in the activity of the enzyme catalyzing the final stage of its biosynthesis, we examined AO activity. The effect of Mo and W treatment on AO activity was studied in 8-day-old barley grown under non-saline and salt stress conditions, separately for leaves (Figure 6A) and roots (Figure 6B).

In leaves, only one AO activity band was observed, which did not change significantly under the influence of salinity in plants grown on water (−Mo, −W), nor did it change under the influence of Mo in either non-saline or saline conditions. In contrast, treatment with W resulted in a significant reduction in AO activity, by 50% under non-saline conditions and by 30% under saline conditions.

Three AO isoforms were observed in the roots. Salinity caused a 25% increase in total AO activity in the roots of plants growing in water. The use of Mo caused an 80% increase in AO activity under non-saline conditions and a 100% increase under saline conditions. In turn, treating plants with W reduced AO activity by about 50% in both non-saline and saline conditions.

Based on the results obtained, it can be concluded that the changes in AO activity observed in plants grown with Mo and W are closely related to changes in AO activity, especially in roots, under both non-saline and saline conditions.

## 3. Discussion

Exposure of plants to salt stress negatively affects their growth, physiological functions, and yields, with the scale of the response depending on the severity of the stress [52,53]. In our study, NaCl at a concentration of 125 mM caused a significant reduction in biomass production (Figure 2) and induced oxidative stress, measured by the content of H_2_O_2_, O_2_^−^, and MDA, in both leaves and roots (Figure 3). It also caused a decrease in chlorophyll content (Figure 3) and an increase in proline concentration, which was associated with the induction of *P5CS1* expression (Figure 4). Overexpression of *P5CS* genes in transgenic Arabidopsis, tobacco, or wheat increases proline content and ultimately tolerance to drought, heat, and salinity [54,55,56]. In turn, the upregulation of *P5CS1* expression in barley was shown to promote stress-inducible proline accumulation in response to drought and cold [22,57]. Furthermore, allelic variations in the barley *P5CS1* gene, arising from mutations in the abscisic acid-responsive element (ABRE) of the *P5CS1* promoter, significantly alter *P5CS1* expression and proline accumulation in response to drought [29], highlighting the ABA-dependent nature of proline synthesis under stress conditions. This was further confirmed by exposing rice to norflurazon, an ABA synthesis inhibitor, which suppressed proline synthesis and intensified oxidative damage caused by hypoxia [58]. Although some researchers reported a positive relationship between endogenous ABA levels and Pro accumulation [59], other studies have shown that proline accumulation under stress may be ABA-independent. In the tomato *flacca* mutant, which lacks AO activity and does not accumulate ABA under osmotic stress, proline accumulation was significantly increased [32]. Increased proline synthesis was also observed in ABA-deficient *aba1-1* mutants, as well as in ABA-insensitive *abi1-1* and *abi2-1* mutants, under cold and osmotic stress [32]. Moreover, in pea plants exposed to Cd toxicity, it was reported that the accumulation of proline precedes the increase in ABA content [19]. Under our experimental conditions, the increase in proline content and *P5CS1* expression in barley growing under salinity was not accompanied by an increase in ABA concentration (Figure 5), supporting the possibility of an ABA-independent mechanism of proline accumulation in response to stress. However, ABA concentration was not assessed dynamically, and it cannot be ruled out that ABA content may have increased in earlier stages of salinity exposure and that after 8 days of the experiment it returned to control values. To further elucidate the mechanism of proline accumulation under stress conditions, we exposed barley simultaneously to NaCl and metals, molybdenum or tungsten, which will be discussed below.

Although under our experimental conditions, treatment with NaCl (−Mo, −W) did not increase ABA levels, a rise in AO activity was already evident (Figure 6). This may suggest that extending the experiment would also lead to higher ABA content or that AO contributes to oxidative stress generated at this stage by NaCl treatment. Previous studies have shown that native AO from Arabidopsis and tomato can produce H_2_O_2_ and may represent a new source of ROS generated under water stress [60].

In recent years, several reports have highlighted the beneficial effects of molybdenum on plant growth and development in response to abiotic stresses [45,46,47,48,49]. However, the molecular and biochemical mechanisms underlying these processes remain poorly understood. The reduction of oxidative stress marker levels by Mo supplementation, observed in wheat under drought conditions [46], rice exposed to cadmium [49], and soybean under saline–alkaline conditions [61], is believed to be at least partly due to increased activity of ROS-detoxifying enzymes such as catalase (CAT), ascorbate peroxidase (APX), and superoxide dismutase (SOD). It has also been suggested that Mo-induced antioxidant protection may be mediated by nitric oxide, whose increased production results from Mo-induced NR activity, as observed in wheat under drought stress conditions [62]. In barley, 0.5 mM Mo significantly mitigated the NaCl-induced decrease in biomass production (Figure 2) and chlorophyll content (Figure 3), and alleviated NaCl-induced oxidative stress (Figure 3) almost to the level of control plants (−Mo, −W), especially in leaves. We hypothesized that Mo may enhance barley tolerance to salt stress by activating Mo-dependent AO activity, which in turn increases ABA synthesis and subsequent ABA-dependent proline accumulation. Previous studies showed that in wheatgrass subjected to salt stress, Mo application significantly improved plant health and protected against oxidative damage, which was believed to be associated with increased activity of the Mo-containing enzymes–AO, XDH, and NR [47], although ABA content was not determined. In Mo-treated barley, we observed not only an increase in AO activity in roots but also a corresponding increase in ABA content in leaves and roots (Figure 5 and Figure 6) under both non-saline and saline conditions, confirming the first part of our hypothesis.

To further verify our hypothesis, we examined proline content and key proline metabolism gene expression in plants exposed to Mo under both non-saline and saline stress conditions. Under non-saline conditions, Mo induced an increase in proline content and *P5CS1* expression (Figure 4) in both leaves and roots, which may have resulted from Mo-induced increases in ABA content (Figure 5). Under saline conditions, proline level and *P5CS1* expression were similar between Mo-treated and non-treated plants, although, as mentioned above, the ABA level was significantly higher in the former. These findings suggest that Mo application reduces NaCl-induced oxidative stress, likely through ABA-dependent mechanisms, but these are not related to proline accumulation. Reports on the effect of molybdenum on proline content are scarce and often contradictory. For example, in rice, Mo treatment decreased proline content under control conditions but increased it under Cd stress [49]. In sorghum, Mo application increased proline content under control conditions, but significantly lowered it under salt stress [63]. In barley, the increase in proline level due to Mo application was accompanied by higher *P5CS1* expression, while Mo had no significant effect on *PDH* expression (Figure 4). To our knowledge, these results are the first to examine the effects of Mo and W on the expression of key proline metabolism genes in plants grown under both non-saline and saline conditions. In summary, the results indicate a positive relationship between Mo application and ABA-dependent proline synthesis, but only under non-saline conditions. Under salt stress, proline synthesis and accumulation seem to depend more on the level of oxidative stress than on the level of ABA. As Mo restores oxidative stress marker levels nearly to those observed under non-saline conditions, there are no differences in proline levels in Mo-treated plants between non-saline and saline conditions.

To confirm that under salinity conditions, proline accumulation depends more on oxidative stress than on ABA levels, we conducted an experiment using tungsten. Exposure of plants to 0.5 mM W caused a significant loss of biomass in both leaves and roots, with more pronounced effects in the roots (Figure 2). Similar results were reported in response to high concentrations of W in pea and cotton seedlings [64], wheat [65], Arabidopsis [66], and soybean [67]. In barley grown under saline conditions, the deleterious effects of W exposure were even more severe than under non-saline conditions (Figure 1 and Figure 2). Under salinity stress, W inhibited Mo-dependent AO activity (Figure 6), and accumulation of stress-induced ABA was not observed (Figure 5), despite a dramatic increase in oxidative stress indicators (Figure 3). Although inhibition of AO activity by W has been previously reported in barley [45,52] and maize [68], this was not always associated with a decrease in ABA content, especially under control conditions [68]. In our experiment, 0.5 mM W also did not affect ABA content under non-saline conditions, which may be related to the fact that free ABA concentration depends not only on de novo synthesis of ABA or its degradation. ABA also occurs in cells in a form conjugated with glucose, as ABA-glucosyl esters [69], from which it is released by specific β-glucosidases, and in this way can compensate for deficiencies resulting from impaired de novo synthesis. However, this compensatory process is not efficient under drought stress, where application of W has an inhibitory effect on stress-induced ABA accumulation [68]. Additionally in our experiment, no increase in ABA content was observed under salt stress with the application of W (Figure 5), while proline content and *P5CS1* expression increased several times under both non-saline and saline conditions, with this effect being more pronounced in the roots (Figure 4). Increased proline content in response to high W concentrations has also been observed in broccoli [70] and wheat [65]; however, the expression of genes involved in proline metabolism or ABA content was not studied. W is a nonessential element, considered toxic to plants [71], as it not only disrupts the function of Mo enzymes but also interferes with cell division, impairs cytoskeletal integrity, and induces programmed cell death [72]. The molecular mechanisms of plant defense against W toxicity are largely unknown [71]; therefore, the results of this study will provide new insights into this area. An increase in proline content under conditions of high tungsten concentration may be particularly important for maintaining effective antioxidant defense. As demonstrated in broccoli seedlings [70] and cereals such as wheat, barley, and oats [73], high concentrations of W led to the collapse of the ROS-metabolizing system, resulting in increased levels of hydrogen peroxide, superoxide anions, and hydroxyl radicals, and consequently, oxidative damage. In these experiments, reduced activity of SOD, CAT, APX, and glutathione peroxidase was observed, along with lower levels of non-enzymatic antioxidants such as ascorbic acid, α-tocopherol, phenolic compounds, flavonoids, and reduced glutathione [70,73]. In our experiment with barley grown under non-saline conditions, we came to similar conclusions when comparing the activity of several antioxidant enzymes. While Mo application induced APX and CAT activity in barley leaves, W supplementation, in turn, significantly reduced the activity of APX and SOD (Figure S1). Therefore, the increase in proline content in barley in response to high W concentrations may compensate for the decline in enzymatic antioxidant defense; however, further research is needed to confirm this hypothesis.

## 4. Materials and Methods

### 4.1. Plant Material and Experimental Conditions

The research was carried out on 8-day-old seedlings of barley (*Hordeum vulgare*) cv. Baisheshek. Before sowing, the seeds were surface sterilized with 50% sodium hypochlorite for 10 min and 70% ethanol for 1 min. The seeds were then rinsed three times with distilled water and placed on two layers of filter paper in glass jars (11 cm × ϕ 9 cm; 20 seeds per jar) and moistened with 10 mL of one of six treatment solutions: water (control), 125 mM NaCl, 0.5 mM Na_2_MoO_4_, 0.5 mM Na_2_WO_4_, 0.5 mM Na_2_MoO_4_ + 125 mM NaCl, or 0.5 mM Na_2_WO_4_ + 125 mM NaCl. For each treatment, five jars were prepared simultaneously, constituting one biological replicate (Figure 1). The seeds were germinated at 23 °C in the dark, then transferred to the light to simulate natural conditions. From that point, the filter paper in the jars was moistened daily with 5 mL of water for each treatment. Eight days after sowing, seedlings were harvested and roots were separated from leaves. Fresh samples were used to determine ROS concentration, while the remaining plant material was frozen in liquid nitrogen and stored at −80 °C until use. Seedlings were grown in a growth chamber (MLR-352H climatic chamber, PHC Biomedical, Tokyo, Japan) under long photoperiod conditions (16 h light/8 h dark), relative air humidity of 60%, day temperature of 22 °C, and night temperature of 19 °C. The experiment was repeated independently three times.

### 4.2. Determination of Chlorophyll and MDA Concentrations

The total chlorophyll content was measured using the dimethyl sulfoxide (DMSO) method, as described by Hiscox and Israelstam [74]. Plant samples (50 mg) were homogenized with 2 mL of DMSO and then incubated for 60 min at 65 °C. The undissolved particles were removed by centrifugation for 10 min at 12,000× *g*, and the absorbance of the supernatant was measured at 649 nm and 665 nm.

The MDA concentration was measured according to the thiobarbituric acid method, as described by Heath and Packer [75]. Plant samples (0.2 g) were homogenized with 2 mL of 10% trichloroacetic acid (TCA) and centrifuged for 15 min at 12,000× *g*. An equal volume of TBA reagent (0.5% thiobarbituric acid in 5% TCA) was then added to the supernatants and the mixtures were incubated for 45 min at 100 °C. Finally, the mixtures were centrifuged for 15 min at 12,000× *g* and the absorbance was measured at 532 nm and 600 nm.

### 4.3. Determination of ROS Concentration

The O_2_^−^ concentration was determined using the method described by Elstner and Heupel [76] and adopted by others [77,78], with some modifications. Plant samples (0.2 g) were ground in 0.8 mL of 65 mM potassium phosphate buffer, pH 7.8, containing 7.5 mM hydroxylamine hydrochloride. The extracts were centrifuged for 15 min at 13,000× *g* and 4 °C. Then, 0.17 mL of extraction buffer and 0.03 mL of 50 mM hydroxylamine hydrochloride were added to 0.1 mL of the resulting supernatants and the mixtures were incubated for 20 min at 25 °C. Next, 0.3 mL of 7 mM N-(1-naphthyl)ethylenediamine and 0.3 mL of 58 mM sulfanilamide were added and the mixtures were incubated again for 20 min at 25 °C. The mixtures were then combined with an equal volume of chloroform and centrifuged for 5 min at 13,000× *g*. The absorbance of the resulting upper phase was measured at 530 nm.

The H_2_O_2_ concentration was measured as described by Jana and Choudhuri [79] and used by others [78]. Briefly, plant samples (0.25 g) were homogenized with 2.5 mL of 50 mM potassium phosphate buffer, pH 6.5, and the extracts were centrifuged for 25 min at 6000× *g*. Next, 0.2 mL of 0.1% titanium chloride in 20% H_2_SO_4_ was added to 0.6 mL of the supernatants, the mixtures were centrifuged for 15 min at 6000× *g*, and the absorbance was measured at 410 nm.

### 4.4. Determination of Proline Concentration

Plant samples (0.2 g) were homogenized with 2 mL of 3% (*w*/*v*) sulfosalicylic acid, shaken for 30 min at 750 rpm, and centrifuged for 20 min at 4 °C and 12,000× *g*. Proline content was then determined as described by Bates et al. [80]. Briefly, the supernatants were mixed with equal volumes of ninhydrin reagent and glacial acetic acid, and the resulting mixture was heated at 100 °C for 1 h in a water bath. The reaction was then stopped in an ice bath and the mixture was extracted with toluene. The optical density of the toluene phase was measured at 520 nm.

### 4.5. Gene Expression Analysis

Total RNA was extracted according to the method of Chomczynski and Sacchi [81], with minor modifications as described by Zdunek-Zastocka et al. [78]. The isolated RNA was treated with DNase I, and 2 µg of RNA was reverse transcribed using the Transcriptor First Strand cDNA Synthesis Kit (Roche Diagnostics, Mannheim, Germany). The resulting cDNA was used for gene expression analysis by real-time PCR with the FastStart Essential DNA Green Master Kit (Roche Diagnostics, Mannheim, Germany). All amplification reactions were conducted on a LightCyclerR 96 instrument (Roche Diagnostics, Mannheim, Germany) under the following cycling conditions: 95 °C for 10 min, 35 cycles of 95 °C for 15 s, 63 °C for 15 s, and 72 °C for 20 s. Melting curves were generated at 95 °C for 10 s, 65 °C for 1 min, and 97 °C for 1 s for each reaction to confirm a single PCR product. All primers used for real-time PCR are listed in Table S1. Genes encoding β-tubulin and actin were used as internal controls. The relative expression levels of target genes were calculated using the 2^−ΔΔCt^ method [82]. All reactions were performed at least in triplicate for each of the three biological replicates.

### 4.6. Quantification of ABA Concentration

Extraction of ABA was performed as described by Zdunek-Zastocka et al. [19]. Briefly, 200 mg of tissue samples were ground with 1.4 mL of extraction buffer containing 80% methanol, 2% glacial acetic acid, and butylated hydroxytoluene (20 mg L^−1^). The homogenates were shaken for 24 h at 4 °C in the dark and then centrifuged twice for 15 min at 12,000× *g* and 4 °C. The resulting supernatants were then diluted 25-fold in Tris-buffered saline (150 mM NaCl, 25 mM Tris-HCl, pH 7.5) and used to determine ABA concentration using the Phytodetek ABA enzyme immunoassay test kit (Agdia, Inc., Elkhart, IN, USA) according to the manufacturer’s instructions.

### 4.7. Aldehyde Oxidase Activity Staining

Plant tissues were ground in prechilled 250 mM Tris-HCl buffer, pH 8.5, containing 10 mM glutathione, 2 mM dithiothreitol, and 1 mM EDTA. The tissue to buffer ratio was 1:2 for leaves and 1:3 for roots (*w*/*v*) [37,83]. The homogenates were centrifuged for 30 min at 12,000× *g* at 4 °C, and the proteins in the resulting supernatants were separated by native polyacrylamide gel electrophoresis (PAGE) on a 7.5% polyacrylamide gel [84]. The amount of soluble protein in the samples was determined according to the Bradford assay [84], with crystalline bovine serum albumin (BSA) as a standard. After electrophoresis, gels were incubated in a reaction mixture consisting of 0.1 M Tris-HCl buffer, pH 7.5, 0.1 mM PMS (phenazine methosulphate), 1 mM MTT (3-(4,5-dimethylthiazol-2-yl)-2,5-diphenyltetrazolium bromide), and 1 mM indole-3-carboxaldehyde at 22 °C in the dark [37,83]. The AO activity was determined based on the intensity of the resulting formazan bands, calculated using ImageJ software version 1.53.

### 4.8. Statistical Analysis

Statistical analyses were conducted on the results of three independent experiments, each with three technical replicates per biological replicate, and are presented as mean ± standard deviation. Data were analyzed using one-way analysis of variance (ANOVA) with Statistica software version 9.1, separately for Mo- and W-treated plants. Data normality was verified with the Shapiro–Wilk test, and homogeneity of variance was confirmed with Levene’s test. Differences between group means were assessed using Tukey’s parametric test.

## 5. Conclusions

Soil salinization poses a serious and growing threat to global agriculture, negatively affecting plant growth and development. Therefore, developing effective technologies to protect plants from the negative effects of high salt concentrations is becoming a priority. One way to reduce the negative impact of chloride salinization may be the use of molybdenum.

In this study, we hypothesized that Mo may increase salt tolerance by inducing AO-dependent ABA biosynthesis, which then leads to ABA-dependent proline synthesis and accumulation. Based on the results obtained, we conclude that molybdenum does indeed induce *P5CS1* expression and proline accumulation, which results from Mo-induced ABA synthesis and accumulation, but only under non-saline conditions. In contrast, under saline conditions, proline levels are similar in Mo-treated and non-Mo-treated plants, despite significantly higher ABA levels in the former. This may suggest the involvement of other ABA-dependent mechanisms in the increased salt tolerance of barley under Mo influence. In turn, the application of W, a Mo antagonist, induced oxidative stress and increased P5S1 expression and proline accumulation in both non-saline and saline conditions, although AO activity was inhibited and, consequently, no increase in ABA content was observed. The results obtained indicate that ABA accumulation is not necessary for proline accumulation under salinity conditions in barley, but is more related to the level of oxidative stress modulated by Mo and W.

## Data Availability

The original contributions presented in this study are included in the article/Supplementary Material. Further inquiries can be directed to the corresponding author.

## Supplementary Materials

The following supporting information can be downloaded at: https://www.mdpi.com/article/10.3390/ijms27021104/s1. References [85,86,87,88] are cited in the Supplementary Material.
