# Supplementary material for: Proline Accumulation in Barley Under Salinity Is ABA-Independent, but Relies on the Level of Oxidative Stress When Modulated by Mo and W Ions
Source: Int J Mol Sci. 2026 Jan 22;27(2):1104. doi: 10.3390/ijms27021104 (PMC12842508; doi:10.3390/ijms27021104)
Supplement: Supplementary file 1 [file ijms-27-01104-s001.zip › ijms-4056627-supplementary.pdf]

Proline accumulation in barley under salinity is ABA-independent, but relies on the level of oxidative stress when modulated by Mo and W ions

Moldir Beisekova, Beata Michniewska, Weronika Kusek, Alua Zh. Akbassova, Rustem Omarov, Sławomir Orzechowski, Edyta Zdunek-Zastocka *

1 Department of Biotechnology and Microbiology, L.N. Gumilyov Eurasian National University, Astana 010000, Kazakhstan; mk.beisekova@gmail.com (M.B.); a.j.alua@gmail.com (A.Z.A.)

2 Department of Biochemistry and Microbiology, Warsaw University of Life Sciences-SGGW, Nowoursynowska 159, 02-776 Warsaw, Poland; beata_michniewska@sggw.edu.pl (B.M.); weronika_kusek@sggw.edu.pl (W.K.); slawomir_orzechowski@sggw.edu.pl (S.O.)

* Correspondence: edyta_zdunek_zastocka@sggw.edu.pl; Tel.: +48 22 593 2577

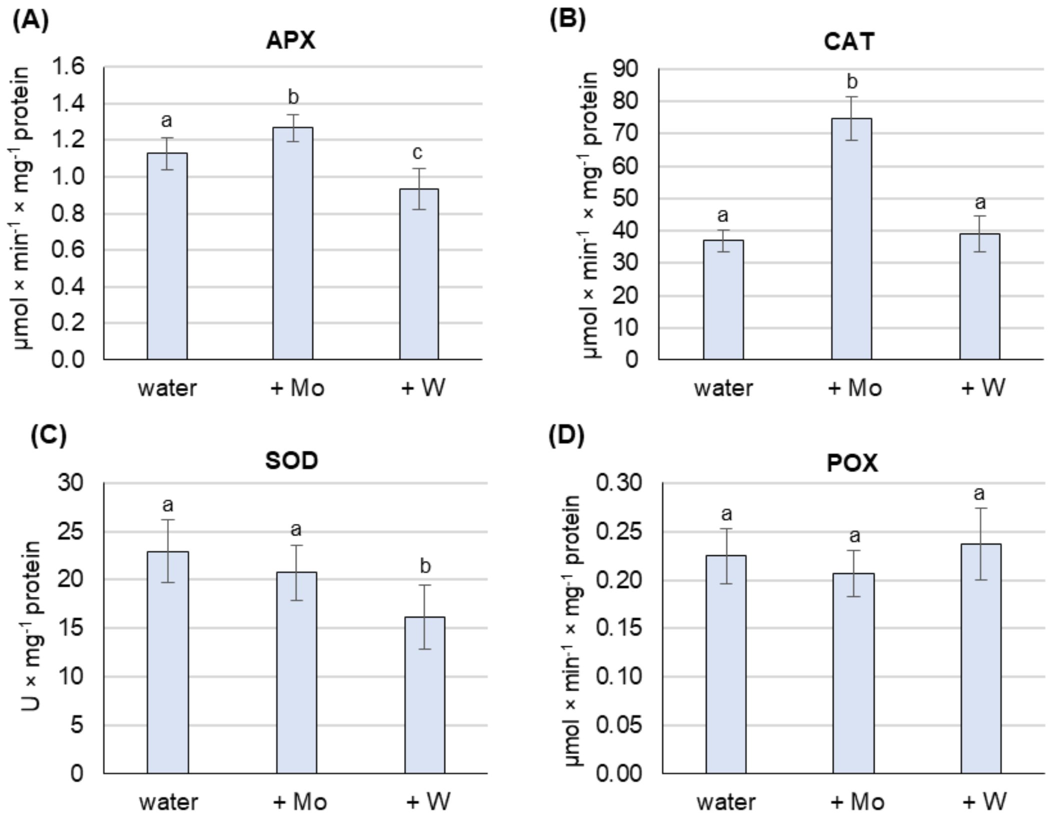

Figure S1. The effect of molybdenum (Mo) and tungsten (W) on the activity of ascorbate peroxidase (A), catalase (B), superoxide dismutase (C), and guaiacol-type peroxidase (D) in leaves of 8-day-old barley grown under non-saline conditions. Mo was applied as 0.5 mM Na_2MoO_4 and W as 0.5 mM Na_2WO_4. APX and CAT activities were examined by monitoring the decrease in absorbance at 290 nm [85] and 240 nm [86], respectively, which resulted from the oxidation of ascorbic acid and the decomposition of H_2O_2. SOD and POX activities were determined based on the inhibition of quercetin oxidation at 406 nm [87] and on the guaiacol oxidation at 470 nm [88], respectively. SOD activity was expressed in arbitary units (the amount of SOD that inhibits superoxide-driven oxidation of quercetin by 50%) per min. Results are the means (±SD) of three biological replicates. Significant differences (at least $p < 0.05$) between the means are marked above the columns with different lowercase letters.

Table S1. Primer sequences used for real-time PCR analysis of the expression of *Hordeum vulgare* genes. F, forward oligonucleotide; R, reverse oligonucleotide.

Gene	Accesion number of the *Hordeum vulgare* sequence in GenBank database	Oligonucleotide sequence (5′->3′)
P5CS1	BK007070	F: GAGGTGATAATGGTCACGTCCGGCGCT<br>R: AGCGCACGCCTTTCCATCCAGCTC
PDH	XM_045115768	F: CGGGATCCTCGACTACGGCATCGA<br>R: GCACAGCGCCGTGATTTTAATACACAC
Ubiquitin	AK357664	F: GCGCAAGAAGAAGACGTACACCA<br>R: GTTGGGGCACTCCTTCCTGAGC
Actin	AY145451	F: GCTGGAGATGATGCGCCAAGGGCT<br>R: CTCTCTTGGACTGCGCCTCATCACCA

References

85. Nakano, Y.; Asada, K. Hydrogen peroxide is scavenged by ascorbate-specific peroxidase in spinach chloroplasts. *Plant Cell Physiol*. **1981**, *22*, 867–880.
86. Aebi, H. Catalase *in vitro*. *Methods Enzymol.* **1984**, *105*, 121–126.
87. Kostyuk, V.A.; Potapovich, A.I. Superoxide-driven oxidation of quercetin and a simple sensitive assay for determination of superoxide dismutase. *Biochem. Int.* **1989**, *19*, 1117–24.
88. Maehly, A.C.; Chance, B. The assay of catalases and peroxidases. *Methods Biochem. Anal.* **1954**, *1*, 357–424.
